# The Importance of Physical Fitness Parameters in Rhythmic Gymnastics: A Scoping Review

**DOI:** 10.3390/sports12090248

**Published:** 2024-09-07

**Authors:** Vasiliki Gaspari, Gregory C. Bogdanis, Ioli Panidi, Andreas Konrad, Gerasimos Terzis, Anastasia Donti, Olyvia Donti

**Affiliations:** 1 School of Physical Education and Sport Science, National and Kapodistrian University of Athens, 17237 Athens, Greece; vgaspari@phed.uoa.gr (V.G.); gbogdanis@phed.uoa.gr (G.C.B.); ipanidi@phed.uoa.gr (I.P.); gterzis@phed.uoa.gr (G.T.); adonti@phed.uoa.gr (A.D.); 2 Institute of Human Movement Science, Sport and Health, University of Graz, 8010 Graz, Austria; andreas.konrad@uni-graz.at

**Keywords:** flexibility, muscle power and strength, coordination, rhythmic gymnasts

## Abstract

This scoping review presents an overview of physical fitness parameters in rhythmic gymnastics as well as the association of fitness with gymnasts’ performance, competitive level, and age. PubMed, Scopus, and Sport Discus databases were searched. Of the 586 records retrieved, 41 studies met the inclusion criteria (n = 1915 participants). The included studies examined flexibility, aerobic capacity, muscle power, muscle endurance, muscle strength, sprint speed, agility, balance, and coordination. Performance was associated with flexibility, aerobic capacity, lower-limb muscle power, agility, muscular endurance, balance, and coordination from a young age. Flexibility, aerobic capacity, and muscle power were, in general, higher in high-level gymnasts than in low-level gymnasts or controls. Older rhythmic gymnasts demonstrated higher scores than the younger ones in flexibility, aerobic capacity, balance, and sport-specific coordination but not in muscle endurance, while some studies reported a decline in muscle power with age. Supplementary physical fitness training improved all physical abilities irrespective of the gymnasts’ level. Rhythmic gymnastics training alone improved muscle power, agility, speed, muscular endurance, and balance to a lesser extent than targeted fitness training. Muscular strength, speed, and agility are largely under-researched in rhythmic gymnastics. Emphasis should be given to targeted strength and power training due to the high mechanical loads placed on skeletally immature athletes.

## 1. Introduction

Rhythmic gymnastics is a sport in which athletes perform individually or in groups of five using hand-held apparatuses (rope, hoop, ball, clubs, and ribbon). It is characterized as an “aesthetic” sport as it combines gymnastics with elements of dance [1]. Artistry and musical interpretation are key performance components of a competitive routine [2]. However, a large part of the performance is dependent on the ability of the gymnast to execute complex movements of high difficulty and risk, including turns, leaps, and acrobatics, while throwing the apparatus several meters into the air. 

Early-sport specialization and high training loads are typical for rhythmic gymnastics, with the aim to improve the technical, aesthetic, and fitness components of the sport [3]. Young rhythmic gymnasts start systematic training during childhood (i.e., 7–8 years) for an average of 18–20 h per week [4], which is increased to 40 h per week in international-level adolescent athletes [5]. Physical conditioning is an integral part of rhythmic gymnastics preparation [4], with flexibility, cardiometabolic fitness, muscle power, balance, and coordination being important performance correlates [6]. Due to the interaction between growth, maturation, and intensive training, the development of these physical fitness parameters in children and adolescent gymnasts is nonlinear and requires careful control of the training load [7]. High and imbalanced training load in growing rhythmic gymnasts may increase the risk of injury [8,9], may have a detrimental effect on skill acquisition and execution due to fatigue [7], and may also increase the risk of overtraining and dropout from sport [10]. In that sense, a balanced and targeted development of key physical fitness parameters may facilitate technical skills learning and athletic development, reduce the risk of injury, and allow gymnasts to tolerate the high demands of training loads and competition [11,12].

Despite the importance of physical fitness for gymnasts’ performance and health across their sports career (i.e., 8–22 years of age), there is limited information in the literature regarding the development and importance of fitness parameters and their impact on performance. Thus, a scoping review was conducted in order to systematically map and summarize the evidence on the physical fitness parameters examined in rhythmic gymnastics, their association with gymnasts’ performance scores, level of performance (e.g., international or national ranking, competitive level, etc.), and age. A further goal was to examine the effectiveness of training interventions, as well as the tests used to assess them. Furthermore, following the principles of scoping reviews, this study aimed to identify research gaps in the literature.

## 2. Materials and Methods

This scoping review aimed to provide an overview of physical fitness parameters in rhythmic gymnastics. A secondary aim was to examine the association of fitness parameters with gymnasts’ performance, competitive level, and age being affected and interrelated with physical fitness. 

This scoping review was designed according to the PRISMA-ScR checklist (Preferred Reporting Items for Systematic Reviews and Meta-analyses Extension for Scoping Reviews) (see supplementary File S1 for PRISMA checklist) and followed the stages of the methodological frameworks of Arksey and O’Malley (2005) [13]. 

### 2.1. Search and Selection Strategy

A literature search was conducted from April 2023 until the end of February 2024 on Scopus, PubMed, and Sport Discus databases by four independent reviewers (V.G., I.P., O.D., A.D.). No study design and date restrictions were applied in the search algorithm. The field types used in the search were “Title”, “Abstract”, and “Keywords”. Additional records were found by searching the reference lists of relevant papers and studies meeting the eligibility criteria and by screening the researchers’ personal lists in Google Scholar. To determine the keywords of the search strategy, the following steps were taken: (a) identification of the research question; (b) identification of related terms for physical fitness; (c) testing of the keywords in relevant databases; and (d) examination of the keywords used in the existing literature. A Boolean search strategy was applied using the following string: ((“rhythmic gymnast*”) AND (flexib* OR power OR strength OR balance OR aerobic OR anaerobic OR speed OR fitness OR coordination) AND (physical OR abilit* OR capacit* OR fitness OR training OR preparation)). Furthermore, one study that was not identified in the systematic search was also included in this review based on our knowledge of the area. Four investigators (V.G., I.P., A.D., and O.D.) selected the eligible studies, and disagreements were resolved by GCB and AK by consensus and discussion with other authors if needed.

### 2.2. Inclusion and Exclusion Criteria

Randomized control trials (RCTs), controlled trials without randomization (CTs), cross-sectional studies (CSs), and longitudinal studies (Ls) examining physical fitness parameters in rhythmic gymnastics were included using the following criteria: (1) articles in peer-reviewed journals in English; (2) studies involving rhythmic gymnastics athletes (non-amateurs); (3) at least one key term of the search string should be included within the title, abstract, or keywords; (4) full text was available, either online or after direct contact with the authors. 

Studies with the following characteristics were excluded: (1) studies that did not provide quantitative data on physical parameters in rhythmic gymnastics; (2) studies including amateur and student athletes; (3) review papers, retrospective studies, case reports, letters to the editor, special communications, invited commentaries, and conference papers. 

### 2.3. Selection of Sources of Evidence

The included papers were imported into Endnote and were examined independently by four authors (V.G., I.P., A.D., and O.D.) in a blinded mode. If necessary, the corresponding authors of the selected articles were contacted by e-mail to request any missing relevant information.

### 2.4. Data Items

Data extraction from the included papers was performed by three independent investigators (V.G., I.P., O.D.) and was supervised by two referee investigators (G.C.B. and G.T.). A data-charting form was jointly developed by the three authors (V.G., I.P., O.D.) to determine which variables to extract. The authors independently charted the data, discussed the results, and continuously updated the data charting. Disagreements were resolved through discussion with the referee investigators (G.C.B. and G.T.). The following data from the included articles were extracted: (1) authors of this paper; (2) date and type of publication; (3) study design type (RCT, CT, CS, LS); (4) sample size, age, and training status of the participants; (5) physical fitness parameter examined; (6) tests used to examine gymnasts’ fitness parameters (general tests or sport-specific); (7) study outcome (e.g., training outcome or association of fitness parameters with performance); and (8) main findings of this study. Gymnast’s level was described as “high” for elite gymnasts, qualifiers in the all-around, National team members, high and intermediate level gymnasts and “low” for initial or club level gymnasts, non-qualifiers in the all-around, low-level gymnasts, and student gymnasts. Because in the eligible studies, “younger” and “older” gymnasts belonged to different age groups (i.e., in some studies, young gymnasts were 8–10 years old, while in other studies, 11–13 years old), it was decided to use the term “younger” and “older” as an age trend and not as a specific age group. The tests used to measure physical fitness were categorized as “specific” or “general-non-specific” based on whether they were specific for the sport (i.e., tests examining technical skills or sport-specific fitness tests) or general fitness tests used for typical athletic population (i.e., countermovement jump, squat jump, etc.). 

## 3. Results

### 3.1. Results of the Search Procedure

Initially, 586 studies were retrieved. After duplicates were removed (n = 154), the remaining studies were screened by title and abstract to identify the relevant studies. Furthermore, one study, which was not identified in the systematic search, was also included in the review based on our knowledge of the area. Overall, 433 studies were screened, and 41 studies met the inclusion criteria and were included in this review. A flow chart of the detailed search process is presented in supplementary File S2.

### 3.2. Characteristics of the Included Studies

The 41 studies included in this review were published between 1997 and 2023 and included 1915 subjects aged 8–24 years, with an average age of 13.7 years. From these studies, seven were RCTs; five were CTs; 26 were CS, and two were LS. Through an additional search of the references and the citations, as well as the inclusion of our own library, one additional paper was identified as being relevant. It should be noted that 16 studies examined more than one fitness component. The fitness parameters examined in this study were flexibility, muscle power and strength, cardiorespiratory fitness, muscular endurance, balance, coordination, speed, and agility. Due to the different methodologies used and the different study designs, no comparison between outcomes was made, and the findings for each fitness parameter were grouped. In addition, the range of evidence is discussed in a narrative format. The findings for each fitness parameter are critically appraised in the following sub-sections. All information about the population, the physical fitness capacity examined, the type of study, the test used, and the main findings of the study are presented in Table 1, Table 2, Table 3, Table 4, Table 5, Table 6 and Table 7.

### 3.3. Flexibility

Flexibility is the ability of a joint or series of joints to move through an unrestricted, pain-free range of motion [14]. Thirteen studies examined flexibility in rhythmic gymnastics [6,15,16,17,18,19,20,21,22,23,24,25,26]. Of these studies, four studies examined differences between higher and lower-level athletes; one study examined differences between age categories; three studies examined the association between flexibility and performance; five studies examined training outcomes; one study compared gymnasts from different countries, and two studies compared gymnasts with athletes from other sports (Table 1). 

In general, increased flexibility in the hip, spine, and shoulders was associated with higher performance scores [15,16,17,18]. High-level gymnasts demonstrated better flexibility scores compared with low-level gymnasts [19,20,21,26] and controls (i.e., non-athletes and athletes from other sports) [22,23], and the same was found for older compared with younger gymnasts [16]. Training interventions were effective in increasing flexibility, except for one study [21] (Table 1).

Specifically, the youngest gymnasts were eight years old, and it was reported that systematic flexibility training started at the age of six. Higher flexibility scores in the hip, spine, and shoulders were associated with higher technical or artistry performance scores [15,16,17,24]. For example, in 9–10-year-old gymnasts, shoulder extensions and sideways leg extensions (développé à la seconde) significantly correlated with the technical execution score [24], and hip flexibility significantly contributed to split leap performance [19]. Notably, the association of flexibility with performance was observed from a very young age and throughout childhood and adolescence [17,24].

Flexibility discriminated high from low-level gymnasts. For example, Douda et al. [6] found that flexibility scores (side split with right leg forward and right leg lift test forward) discriminated elite from non-elite adolescent rhythmic gymnasts. In the same study, flexibility explained 12.1% of the variance of the total score in the all-around (sum of the scores of each apparatus) [6]. Donti et al. [24] found significant differences between qualifiers and non-qualifiers in the all-around competition, in shoulder flexion, straight leg raise, and sideways leg extension. In that study, it was also reported that sideways leg extension, body fat, and push-ups accounted for a large part of the variance in the technical execution score for the non-qualifiers, while for the qualifiers, only sideways leg extension and spine flexibility accounted for the variance in the technical execution score. The results of that study suggest that at a lower level of performance, more physical fitness parameters may have an effect on the technical execution, while at a higher level of performance, where fitness is developed above a certain degree, the gymnast is able to execute a technical routine with less physical effort, and the technical execution score depends more on technical skill competence and stability and less on increased flexibility [24]. As expected, in the one study examining age differences, older gymnasts scored higher than younger in trunk hyperextension [16], probably due to their longer training background. 

As expected, rhythmic gymnasts aged 8–17 years were more flexible than controls in sit and reach and shoulder flexibility measurements [26]. Following 6 months of intervention, rhythmic gymnasts improved their passive and active ranges of motion of the hip and shoulder flexion. In contrast, the hip and shoulder range of motion in the control group did not increase [26]. Flexibility training was, in general, effective in enhancing range of motion. For example, following 48 weeks of intervention, five different fitness programs were found efficient in improving flexibility, with no difference observed between training groups [20]. Nevertheless, one study failed to detect differences between athlete groups, probably due to the short training intervention period (8 weeks) in athletes already trained in flexibility [21]. Notably, resistance training was not effective in improving the flexibility in rhythmic gymnasts, underpinning the need for specific training [18]. 

Between rhythmic gymnasts and controls or athletes with different flexibility training backgrounds (i.e., volleyball athletes), both adult and child rhythmic gymnasts demonstrated greater shoulder, hip, and ankle ranges of motion [22,23]. In addition, in the two studies examining gastrocnemius medialis muscle architecture in adults (20–24 years old) and youth (8–9 years old), rhythmic gymnasts compared to same-age athletes from weight-bearing sports, it was found that adult rhythmic gymnasts had longer gastrocnemius medialis fascicles at rest and during stretching, and the authors concluded that long-term, intensive flexibility training might induce longitudinal fascicle growth [22,23]. In one longitudinal observation, flexibility scores improved over the season [15].

**Table 1:** Studies examining flexibility.

			Participants							
Authors	Joint Examined Active/Passive	Type of Study	EG (n)	CG (n)	Age (EG)	Age (CG)	Level	Tests Used/Specific-Non-Specific	Study Outcome	Main Findings
Aji-Putra et al. (2021) [19]	Hip/Active	CS	32 Q and 20 NQ		10.63 ± 2.9		Low–High	Right and left legs straight ahead; right and left legs straight back/NS	Comparison between gymnasts’ levels.	There was a significant difference between qualifiers and non-qualifiers in front right leg raise back and left leg raise.
Batista et al. (2019) [25]	Hip, trunk, shoulder Active/Passive	CS	9 BNT		20.8 ± 1.9		High (National team)	Leg up with help of the hand; leg up without help of the hand; trunk lift forward; stand-and-reach; rotation of the upper limbs/S-NS	Comparison between gymnasts from different countries.	No difference was found in flexibility tests between the gymnasts of the National teams of Portugal and Brazil. Functional asymmetries in the flexibility tests were found in 88.9% and 50% of Brazilian and Portuguese gymnasts, respectively.
			4 PNT		15.8 ± 1.3					
Batista Santos et al. (2015) [15]	Hip Active/Passive	LS	5		13.60 ± 0.245		High	Supported LL hold to the front; supported LL hold to the side; unsupported LL hold to the front; unsupported LL hold to the side; supported LL hold to the rear; unsupported LL hold to the rear–Penché; splits on two benches/S	Training outcome—longitudinal observation.	The gymnasts showed high levels of active and passive flexibility for the preferred lower limbs but lower levels of the non-preferred lower limbs. However, improvements were observed in the flexibility levels of the non-preferred lower limbs over the season.
Boligon et al. (2015) [16]	Hip, Trunk/Active	CS	10	10	8–10	11–12	High–Intermediate	Split, trunk hyperextension/S	Comparison between gymnasts’ levels and age categories.	Split scores were higher in intermediate-level gymnasts compared to low-level gymnasts, irrespective of age. Trunk hyperextension was also higher in intermediate compared to lower-level gymnasts. In addition, trunk hyperextension was also higher in older gymnasts compared to younger gymnasts.
Donti et al. (2016) [24]	Hip, trunk, shoulder Active/Passive	CS	24 Q		10.2 ± 1.0		Low–High	Shoulder flexion with a wooden stick; sit-and-reach; straight leg raise range; sideways leg extension; bridge/S/NS	Comparison between gymnast’s levels.Association of fitness with performance.	There were significant differences between qualifiers and non-qualifiers in shoulder flexion, straight leg raise, and sideways leg extension.
			22 NQ		9.7 ± 1.5					
Donti et al. (2019) [22]	Ankle/Passive	CS	10 RG		21.3 ± 1.6		High	Standing calf stretching/NS	Comparison between different sports.	During the 1 min static stretching, RG athletes displayed greater fascicle length at rest and during stretching, greater maximal ankle dorsiflexion, and muscle–tendon junction displacement than volleyball athletes.
			10 volleyball athletes		24.3 ± 4.7					
Douda et al. (2007) [26]	Hip, shoulder, trunk Active/Passive	CT	71 RG		8–10, 11–12 13–14 15–17		Low–High	Sit-and-reach test; shoulder flexibility with a yardstick; side splits with right and left leg forward (cm); forward leg lift tests (battement devant); sideward (battement à la seconde) with the right and left legs (°)/S-NS	Training outcome.	RG athletes aged 8–17 were more flexible than controls in sit-and-reach and shoulder flexibility measurements. Following 6 months of intervention, RG athletes improved passive and active ROM of the hip and shoulder flexion. In contrast, hip and shoulder ROM in the CG did not increase.
			81 non-gymnasts							
Douda et al. (2008) [6]	Hip, shoulder Active/Passive	CS	15 elite		13.41 ± 1.62		Low-High	Sit-and-reach test (cm); shoulder flexibility with a yardstick (cm); side splits with right and left leg forward (cm); forward leg-lift tests (battement devant); sideward (battement à la seconde) with the right and left legs (°)/S-NS	Comparison between gymnast’s levels.Association with performance.	Elite athletes demonstrated higher values in side-split and right/ left leg sideways. Sit-and-reach test and left forward leg lift test correlated with performance in elite athletes, while no correlation was found in non-elite athletes between flexibility tests and performance.
			19 non-elite							
Kritikou et al. (2017) [17]	Hip, trunk, shoulder Active/Passive	CS	46		9.9 ± 1.3		High	Shoulder flexion with a wooden stick; sit-and-reach test; “bridge” test/S-NS	Association with performance.	Multiple regression analyses revealed that sideways leg extension and high-intensity shuttle run accounted for 43.7% of the variance of the score of artistry.
Panidi, et al. (2019) [23]	Ankle/Passive	CS	10 trained		8–10		Low–High	Standing wall calf stretch/NS	Comparison between different sports.	Greater fascicle elongation at the mid-belly and the distal part of gastrocnemius medialis during static stretching and greater ankle angles at rest and during dorsiflexion were observed in RG compared to volleyball female athletes. Ankle dorsiflexion significantly correlated with fascicle elongation in gastrocnemius medialis.
			6 not trained							
Piazza et al. (2013) [18]	Hip/Active	RCT	19(RG; UN)		12.0 ± 1.8		-	Active hip abduction; hip external rotation; hip internal rotation/NS.	Training outcome.	After 6 weeks of resistance training, no significant differences were detected among groups for flexibility.
			18(RG; SP)		11.9 ± 1.0					
Rutkauskaitė and Skarbalius (2012) [21]	Hip, trunk Active/Passive	CT	5 (A)		14.4 ± 0.55 A		-	“Bridge” test; “Splits” test; “Leg keeping’’/S.	Training outcome.	No differences were found following 48 weeks of intervention in flexibility tests.
			5 (B)		14.2 ± 0.84 B					
Rutkauskaitė and Skarbalius, (2009) [20]	Hip, trunk Passive	CT	5 (A)		11–12		-	“Bridge” test; “Splits” test/S.	Training outcome.	Following 48 weeks of intervention, five different fitness programs were efficient in improving flexibility, with no difference observed between training groups.
			5 (B)							
			5 (C)							
			5 (D)							
			5 (E)							

Note: S, specific; NS, non-specific; RCT, randomized controlled trial; CT, controlled trial; LS, longitudinal study; CS, cross-sectional study; BNT, Brazilian National Team; PNT, Portugal National Team; CG, control group; EG, experimental group; RG, rhythmic gymnastics; A, group A; B, group B; C, group C; D, group D; E, group E; Q, qualifiers; NQ, non-qualifiers; LL, left leg; UN, unspecific resistance training group; SP, specific resistance training group.

Both passive and active flexibility scores are important for rhythmic gymnasts. Although high and similar values were observed between active and passive flexibility in the hip joint [17,24], spine flexibility tests report a large difference between passive (i.e., “bridge” test) and active flexibility [24], a finding that may partly explain the increased injury and re-injury risk of the lumbar spine [4].

To assess flexibility, passive and active flexibility tests were used. Shoulder, hips, and spine were typically assessed using general tests (i.e., sit and reach) and sport-specific tests (i.e., splits, “bridge”) [6,15,17,25]. General flexibility tests (i.e., sit-and-reach) are valid and reliable measures of range of motion; however, they may not be “sensitive” enough to detect small changes between gymnasts’ levels. For example, Donti et al. [24] reported that from a battery of general and specific tests, the “sit-and-reach” test did not discriminate qualifiers from non-qualifiers in the all-around. In contrast, sideways leg extension was the only flexibility test that predicted technical performance in 9–10-year-old rhythmic gymnasts, underpinning the importance of the testing protocol being biomechanically and physiologically specific to this sport [24]. 

### 3.4. Aerobic Capacity

Aerobic capacity refers to the maximum amount of oxygen a body can use at one time during an intense exercise [27]. Eight studies [6,21,27,28,29,30,31,32] examined aerobic capacity in rhythmic gymnasts. Four studies reported more than one outcome. Seven studies examined the association between aerobic capacity and performance scores; three studies examined differences between performance levels; one study examined cardiorespiratory fitness among different apparatuses (i.e., hoop, ribbon, ball); one study compared elite rhythmic gymnasts with ballet dancers, and one study compared different age categories (Table 2).

Although a large part of daily training in rhythmic gymnastics involves light-intensity training activities, aerobic fitness is a key performance correlate from a young age (8–10 years old) [27]. In general, elite athletes presented higher maximal oxygen uptake values (${VO}^{2}$max) compared to non-elite [6,28,29]. In addition, adolescents showed higher levels of aerobic fitness than preadolescent gymnasts [27], while no differences were found in ${VO}^{2}$max between gymnasts and ballet dancers [30]. 

Douda et al. [28] found that elite gymnasts presented higher ${VO}^{2}$max values at a maximal effort than non-elite, while heart rate and lactate values were similar between elite and non-elite gymnasts. Later, Douda et al. [6] also reported that elite athletes had higher values in ${VO}^{2}$max and heart rate response compared with non-elite. ${VO}^{2}$max (mL_min), heart rate, ventilation (Lmin), $O^{2}$pulse (mL), and exercise time (min) during a graded maximal test were significantly correlated with performance scores in both elite and non-elite athletes [6]. Similar findings on the association between aerobic fitness and performance were also reported by Rutkauskaitė and Skarbalius [21], while the findings of Montosa et al. [27] confirm this association from a young age (8–10 years old). 

Notably, older gymnasts outperformed younger ones in aerobic fitness, when ${VO}^{2}$max was expressed relatively to body mass. Montosa et al. [27] evaluated the cardiorespiratory fitness (${VO}^{2}$expressed in ml/kg/min) in gymnasts of different age categories (i.e., 8–12 vs. 13–17 years) and found that the 8–12-year-old group had lower aerobic fitness than the 13–17-year-old group, probably due to their longer training backgrounds. In another study, high-level gymnasts demonstrated higher ${VO}^{2}$max compared to high-level dancers [30]. A more recent study [31] analyzed training intensity in nine rhythmic gymnasts (20.8 ± 1.9 years old) by means of accelerometers and reported that 35% of the training session was performed at moderate to very vigorous intensity exercises, while 65% of training time included low-intensity activities.

Some studies conducted laboratory tests in combination with field tests or competitive routines to assess aerobic and anaerobic fitness parameters in rhythmic gymnasts [21,30], while no sport-specific tests are reported in the literature.

**Table 2:** Studies examining aerobic capacity.

		Participants							
Authors	Type of Study	EG (n)	CG (n)	Age (EG)	Age (CG)	Level	Test Used/Specific–Non-Specific	Study Outcome	Main Findings
Baldari C. and Guidetti L. (2001) [30]	CS	12 RG athletes;		14.3 ± 1.2		Low–High	Aerobic power (${VO}^{2}$max); individual ventilatory; anaerobic thresholds NS	Comparison between different sports (gymnasts-dancers).	${VO}^{2}$ expressed in mL × kg^−1^ × min ^−1^ was higher in RG athletes compared with dancers and sedentary subjects. Mean blood lactate values were similar in athletes compared with dancers and sedentary subjects. In addition, anaerobic threshold heart rate was higher in RG.
		8 ballet dancers		14.4 ± 1.7					
		12 sedentary		14.1 ± 1.1					
Batista et al. (2018) [31]	CS	9		20.8 ± 1.9		High (National team)	Training intensity: accelerometers/NS	Association with performance.	Data were collected using accelerometers during the basic preparatory period of the year. It was observed that 35% of the training session was composed of moderate to very high-intensity exercises, while 65% of the training included light-intensity training activities.
Douda et al. (2006) [28]	CS	15 elite		13.07 ± 1.6		Low–High	*VO^2^* max on a cycle ergometer; blood lactate RPE/NS	Comparison between gymnasts’ levels.Association with performance.	Elite gymnasts presented higher RPE and *VO^2^* max values at a maximal effort than non-elite, while heart rate and lactate values were similar between elite and non-elite gymnasts. Significant correlations were found between RPE values and athletic performances in both elite and non-elite gymnasts.
		24 non-elite							
Douda et al. (2008) [6]	CS	15 elite		13.41 ± 1.62		Low–High	Cycle ergometer up to exhaustion/NS	Comparison between gymnasts’ levels. Association with performance.	Elite athletes had higher values in *VO^2^* max and heart rate response compared with non-elite. *VO^2^* max (mL_min), HR, ventilation (L_min), O_2_ pulse (mL), and exercise time (min) significantly correlated with performance scores in both elite and non-elite athletes.
Guidetti et al. (2000) [32]	CS	9		13–16		High	Maximal continuous treadmill test. 90 s rhythmic ball-routines S/NS	Association with performance.	During ball routine, the peak HR was 188 ± 5 beats/min corresponded to 93.5% of treadmill HRmax. HR and ${VO}^{2}$ expressed in absolute values showed high and significant correlations (*p* < 0.001) during pre-exercise (*r* = 0.86), exercise (r = 0.95), and fast recovery (*r* = 0.98), while a low and not significant correlation was shown during slow recovery (*r* = –0.33). Exercise intensities in ball and treadmill expressed as HRi % and % of ${VO}^{2}$max were similar and not significantly different. The most important energy source during ball routine was the aerobic source. The high anaerobic threshold found in this study enabled gymnasts to perform this high-intensity work with relatively low levels of blood lactic acid.
Manos et al. (2012) [29]	CS	10		15–17		High	Oxygen consumption NS	Association with performance. Comparison between gymnasts’ levels and apparatuses.	Oxygen consumption and several physiological indicators were compared among laboratory, training, and simulated competitive conditions. The mean lactate and its peak were higher during competitive conditions. Higher lactacidemia was observed in the gymnasts of the main team compared with substitute gymnasts. Higher lactacidemia was also observed in the group event of 2 hoops and 3 ribbons compared to the 5-ball group routine.
Montosa et al. (2018) [27]	CS	116		8–12 (n = 56) 13–17 (n = 60)		-	Twenty m shuttle runs NS	Comparison between gymnasts’ age categories. Association with performance.	In the total sample, 13.8% and 23.3% of the gymnasts presented very high and high aerobic capacities, respectively. Significant differences were found between the two age groups (children and adolescents) in ${VO}^{2}$max, with adolescent gymnasts presenting higher aerobic capacity than child gymnasts. Aerobic capacity in adolescent gymnasts correlated with BMI and weight.
Rutkauskaitė and Skarbalius (2012) [21]	CT	5 (A)		14.4 ± 0.55 (A)		-	Maximal oxygen consumption (treadmill test, heart rate measurement, blood samples)	Association with performance.	Aerobic fitness was significantly associated with sports performance in 14–15-year-old gymnasts.
		5 (B)		14.2 ± 0.84 (B)					

Note: S, specific; NS, non-specific; RCT, randomized controlled trial; CT, controlled trial; LS, longitudinal study; CS, cross-sectional study; CG, control group; EG, experimental group; A, group A; B, group B; RG, rhythmic gymnastics; *VO^2^* max, maximal oxygen consumption; RPE, rating of perceived exertion; HR, heart rate; BMI, body mass index.

### 3.5. Muscle Power and Strength

Muscular strength is the ability to exert maximal force in one single contraction [33]. Muscular power refers to a great force production over a short period of time, such as in fast leg kicks and explosive jumping [34]. Nineteen studies examined muscle power [6,17,18,19,20,21,24,25,26,35,36,37,38,39,40,41,42,43,44]. Seven studies examined training outcomes; five studies examined the association between muscle power and performance; seven studies compared differences between performance levels; three studies examined differences according to age categories; and one study compared gymnasts from different countries (Table 3).

Lower-limb muscle power was associated with performance in most, but not all, studies included in this review. In general, high-level gymnasts demonstrated higher lower-limb muscle power compared to low-level gymnasts [19,35,36,37], and the same was found for older gymnasts compared with younger [25,26] and for gymnasts compared with non-athletes [19,35,36,37]. Training interventions improved muscle power and jump height and decreased ground contact time in the hopping test [1,18,20,21,38,39,40].

It was found that lower-limb muscle power is, in general, an important performance determinant for rhythmic gymnastics. For example, Di Cagno et al. [36] examined the association between vertical jumping (countermovement jump and hopping test) and sport-specific technical leaps (i.e., split leaps with stretched legs, ring leap, backward trunk bend leap) and found that ground contact time of the hopping test was significantly correlated with split leaps (r = 0.613, *p* < 0.01), ring leaps (r = 0.632, *p* < 0.01), and backward trunk bend leaps (r = 0.542, *p* < 0.01) when all the gymnasts were examined together (elite and sub-elite) [36] and that the height of hopping test was significantly higher in elite than sub-elite gymnasts, probably showing more effective use of the stretch-shortening cycle in elite gymnasts. Similarly, standing long jump was associated with split leap score [19], and explosive strength was significantly associated with performance in 14–15-year-old rhythmic gymnasts [21]. Nevertheless, in the study of Douda et al. [6], no differences were found in standing long jump and vertical jump performance between elite and non-elite athletes, and no correlations were found between these variables and rhythmic gymnastics performance in both elite and non-elite athletes. Collectively, it seems that lower-limb muscle power discriminates athletes’ levels and is associated with technical skill execution but not always with comprehensive measures of performance, such as an all-around score. 

Lower-limb muscle power increases with age. In the study of Gateva [41], vertical jump height was higher in older gymnasts (19 years old) compared to younger gymnasts (11 years old), while no differences were observed among gymnasts aged 12–17. Importantly, in the study of Cicchella et al. [42], all jumps normalized to Body Mass Index declined with age due to an increase in body size without simultaneous gains in muscle power. 

Training interventions lasting from 4 to 48 weeks were effective in increasing muscle power in children’s and adolescents’ rhythmic gymnasts [18,26,38,39,40]. Although non-specific weight training protocols also increased jumping performance in rhythmic gymnasts, only specific weight training improved hopping test ground contact time, a fitness parameter that was strongly associated with leaping ability [18]. Notably, rhythmic gymnastics training alone was also effective in improving lower-limb muscle power, albeit to a lesser extent than additional weight or resistance training, showing the importance of including developmentally appropriate physical fitness training in developing gymnasts. 

General (countermovement jump, drop jump, hopping test, standing long jump) and specific tests (e.g., ring leap, split leap, etc.) were used in the included studies, and their association with performance was significant in most studies. However, the tests that discriminated high from low-level rhythmic gymnasts were specific (e.g., hopping test, ground contact time, rope skipping, and partial trunk elevation) [25,35].

Regarding muscle strength, only two strength studies were retrieved [45,46]. One study compared muscular strength between gymnastics sports (i.e., artistic, rhythmic, aerobic), and the other examined the effect of 12 weeks of core training on core muscular performance and balance (Table 3). In one intervention study, total body, trunk lean mass, bone mass, and the values of isometric core strength and endurance increased following 12 weeks of core training [45]. The second study examined variables of dynamic balance and isokinetic leg muscle strength among rhythmic, aerobic, and artistic gymnasts. Average power produced during knee extension and knee flexion at 60°/s, 180°/s, and 300°/s differed significantly among the three groups. Correlations were found between the composite score of the Y-balance test and isokinetic strength, showing that muscle strength contributes to dynamic balance [44]. Obviously, muscle strength is largely under-researched in rhythmic gymnastics. This finding is very important because rhythmic gymnastics training and competition place extremely high mechanical loading on skeletally immature athletes, who possibly have sub-optimal strength levels.

**Table 3:** Studies examining muscle power and strength.

		Participants							
Authors	Type of Study	EG (n)	CG (n)	Age (EG)	Age (CG)	Level	Test Used/Specific-Non-Specific	Study Outcome	Main Findings
Muscle power									
Agostini et al. (2017) [43]	RCT	15	15	15.4 ± 1.2	15.2 ± 1.5	Low–High	Vertical jump; horizontal jump/NS	Training outcome.	Following a 12-month intervention, vertical jump and horizontal jump were improved in the EG and the CG, with a larger improvement observed in the EG compared with the CG.
Aji-Putra et al. (2021) [19]	CS	32 Q and 20 NQ		10.63 ± 2.9		Low–High	Split jump; long jump/S-NS	Comparison between gymnasts’ levels. Association with performance	There was a significant difference between qualifiers and non-qualifiers in the split leap score and the standing long and vertical jumps. Long jump performance significantly correlated (*p* = 0.01) with split leap score only in the qualifiers.
Batista et al. (2019) [25]	CS	9 BNT		20.8 ± 1.9		High (National Team)	Front power kicks; back power kicks; partial trunk elevations; partial curl-ups; rope skipping CMJ/NS-S	Comparison between different countries.	Brazilian gymnasts demonstrated better results in almost all the strength tests compared with Portuguese gymnasts.
		4 PNT		15.8 ± 1.3					
Batista et al. (2019) [44]	CS	84 beginners		13.5 ± 2.3		Low–High	Front power kick Back power kick Partial Trunk Elevations Partial Curl-Ups Rope skipping Vertical Jump/NS-S	Comparison between gymnasts’ levels.	Elite and 1st division gymnasts demonstrated higher results than initial-level gymnasts in all the muscle power tests. In addition, elite-level gymnasts score higher than 1st division gymnasts in three out of six power tests that were used in this study.
		71 1st division		13.6 ± 2.1					
		9 elite		14.8 ± 1.8					
Batista et al. (2017) [35]	CS	68		11.7 ± 0.6		Low–High	Front power kicks; back power kicks; partial trunk elevations; partial curl-ups; rope skipping CMJ/NS-S	Comparison between gymnasts’ levels.	Gymnasts from the 1st division presented higher results compared with initial-level gymnasts in all the power tests. The same was found for the finalists of the 1st division and initial level gymnasts compared with gymnasts, who did not enter the finals. Notably, the “rope skipping” and “partial trunk elevations” tests were the power tests that best discriminated gymnasts.
Battaglia et al. (2014) [38]	RCT	36	36	13.8 ± 1.3	14.2 ± 1.7	High (National Team)	HT DJ CMJ/NS	Training outcome.	Before and after six weeks of training, jumping performance was measured in the EG (video observation, mental training, and physical practice) and the CG (physical practice only). Compared with the CG, the EG demonstrated higher flight time in CMJ, DJ, HT and shorter contact time in HT, DJ. In the CG, flight time and contact time of DJ also improved following six weeks of typical rhythmic gymnastics training.
Cicchella et al. (2019) [42]	CS	40		12.4 ± 1.8		High	SJ CMJ, CMJ with arms swinging/NS	Association with performance. Comparisons between gymnasts’ age.	Anatomical cross-sectional area of the thigh correlated with jump height and age and showed a decline with age. All jumps normalized to Body Mass Index declined with age due to an increase in body size without simultaneous gains in strength. The difference between CMJ and SQJ (elasticity) increased from the age of 12 and was higher in older RG athletes.
Di Cagno et al. (2008) [36]	CS	8 elite		14.7 ± 2.2		Low–High	CMJ HT technical split leaps with stretched legs, with ring, and with back bend of the trunk/S-NS	Comparison between gymnasts’ levels. Association with performance.	HT height was higher in elite than sub-elite gymnasts, but no significant differences were found between the two groups in HT ground contact time and CMJ height. In addition, no significant differences were found between groups in the technical split leap parameters. HT ground contact time was significantly correlated with performance and was the best predictor of technical split leaps performance when all the gymnasts were studied together.
		17 sub-elite							
Dobrijević et al. (2018) [39]	RCT	43	31	8 ± 0.8		Low	CMJ Standing long jump test/NS	Training outcome.	Following 12 weeks of proprioceptive training, the EC demonstrated improvements in lower leg muscle power. In contrast, no improvements were observed in CG.
Donti et al. (2016) [24]	CS	24 Q		10.2 ± 1.0		Low–High	CMJ DJ/NS	Comparison between gymnasts’ levels.	There were no significant differences between qualifiers and non-qualifiers in lower-limb muscle power (CMJ height).
		22 NQ		9.7 ± 1.5					
Douda et al. (2007) [26]	CT	71 RG		8–10, 11–12 13–14 15–17		Low–High	Standing long jump; vertical jump/NS	Training outcome. Comparison between gymnasts’ age categories.	RG athletes attained better scores in jumping ability compared to CG before intervention. Following 6 months of training, vertical jump improved in the RG athletes, but not standing long jump. Improvements were also observed in the CG in standing long jump and vertical jump. Sprint speed was better in RG compared to controls but reached a plateau after the age of 11. Younger gymnasts (8–10 years) scored higher in sit-ups than older gymnasts. All 10–14 years old gymnasts showed a rapid improvement in standing long jumps.
		81 non-gymnasts							
Douda et al., (2008) [6]	CS	15 elite 19 non-elite		13.41 ± 1.62		Low–High	Standing long jump; vertical jump/NS	Comparison between gymnasts’ levels.	No differences were found in standing long jumps and vertical jumps between elite and non-elite athletes. No correlations were found between these variables and performances in both elite and non-elite athletes.
Gateva, (2013) [41]	CS	120		11–19.		Low–High	Vertical jump with free arms/NS	Comparison between gymnasts’ age categories.	Vertical jump height was higher in older gymnasts (19 years old) compared to younger gymnasts (11 years old), while no differences were observed among gymnasts aged 12–17 years old. Correlations were found between back muscles and lower limb power.
Hutchinson et al. (1998) [40]	CT	6	2	15–17		Low–High	Leaping test/S	Training outcome.	Following 4 weeks of a specific leaping protocol training, leap height, ground reaction time, and explosive power improved in the EG of elite RG. No differences were observed in the CG. Gains in jumping ability were maintained for 4 months and 1 year post-training.
Kritikou et al. (2017) [17]	CS	46		9.9 ± 1.3		High	CMJ DJ/NS	Association with performance	CMJ and drop jump did not correlate with artistry performance in young RG athletes.
Kums et al. (2005) [37]	CS	11 gymnasts	15 controls	12.7 ± 1.7	12.7 ± 0.7	Low–High	SJ CMJ DJ/NS	Comparison between gymnasts’ levels.	Jump height in SJ, CMJ, and DJ were greater in RG than CG. Jump height in DJ was greater compared with SJ and CMJ only in RG. The ratio of CMJ: SJ height did not differ between RG and CG, while the ratio between drop jump: squat jump was greater in RG compared with CG.
Piazza et al. (2013) [18]	RCT	19 (RG; UN)		12.0 ± 1.8		-	SJ CMJ HT/NS	Training outcome.	Both unspecific and specific weight training protocols increased jumping performance in the two groups. Higher increases were also observed in HT flight time and CMJ flight time following unspecific weight training, while HT ground contact time improved only following specific weight training.
		18(RG; SP)		11.9 ± 1.0					
Rutkauskaitė and Skarbalius, (2012) [21]	CT	5 (A)		14.4 ± 0.55 A		-	Standing long jump on both feet/NS	Association with performance.	Explosive strength was significantly associated with sports performance in 14–15-year-old gymnasts. Following intervention, significant improvements were found in explosive strength.
		5 (B)		14.2 ± 0.84 B					
Rutkauskaitė and Skarbalius, (2009) [20]	CT	5 (A)		11–12		-	Standing long jump on both feet/NS	Training outcome.	Following 48 weeks of intervention, five different fitness programs were efficient in improving muscular explosive strength, with no difference observed between training groups.
		5 (B)							
		5 (C)							
		5 (D)							
		5 (E)							
Muscle Strength									
Esteban-García et al. (2021) [45]	RCT	12	12	13.50 ± 3.17	14.41 ± 2.35	High (National level)	Isometric strength of trunk NS	Training outcome.	The EG improved body composition, trunk lean mass, lean mass, and bone mass following 12 weeks of training. The core training protocol increased isometric strength of trunk, flexion test, and extension test.
Kyselovičová et al. (2023) [6]	CS	6 RG		RG 17.7 ± 0.53		High	Isokinetic leg muscle strength test NS	Different sports.	Average power produced during knee extension and knee flexion at 60°/s, 180°/s, and 300°/s differed significantly among rhythmic, aerobic, and artistic gymnasts. Correlations were found between the composite score of the Y-balance test and isokinetic strength.
		5 AR		AR 14.4 ± 5.92					
		7 AE		AE 15.87 ± 0.73					

Note: S, specific; NS, non-specific; SJ, squat jump; CMJ, countermovement jump; DJ, drop jump; HT, hopping test, RCT, randomized controlled trial; CT, controlled trial; CS, cross-sectional study; BNT, Brazilian National Team; PNT, Portugal National Team; CG, control group; EG, experimental group; RG, rhythmic gymnastics; A, group A; B, group B; C, group C; D, group D; E, group E; UN, unspecific resistance training; S, specific resistance training; AR, artistic gymnastics; AE, aerobic gymnastics; Q, qualifiers; NQ, non-qualifiers.

### 3.6. Sprint Speed and Agility 

Sprinting is running over a short distance at the topmost speed of the body in a limited time [47]. Only three studies examined sprint speed in rhythmic gymnasts [6,26,47]. Three studies compared sprint speed between gymnasts’ levels; one study examined a 6-month intervention, and the last study examined differences in several speed parameters between different age groups (Table 4). 

Douda et al. [6] compared sprint speed between elite and non-elite athletes and reported no differences between gymnasts’ levels. In addition, gymnasts significantly improved their sprint speed in longer distances compared to shorter distances (i.e., 10 vs. 30 m), although after the age of 11, a plateau was observed in sprint performance time. Ivanova [47] examined preadolescent gymnasts (10–12 years old) in different sprint speed and agility tests compared to controls (physically active children) and found that the experimental group outperformed the control only in sport-specific parameters (i.e., temping test, zig-zag test) and not in sprint speed. The authors concluded that insufficient attention is paid to sprint speed training in rhythmic gymnastics [47]. Rhythmic gymnastics training alone also improved sprint speed but to a lesser extent than training intervention [26]. 

Agility has been defined as a rapid and accurate whole-body movement with a change in velocity, direction, or movement pattern in response to a stimulus [24]. Regarding agility, four studies examined this parameter in rhythmic gymnastics [17,24,39,43]. One study compared elite to non-elite gymnasts [24]; one study examined the effect of proprioceptive training intervention on agility [1,39], and another study examined the influence of 12 months of plyometric training in addition to rhythmic gymnastics training on agility [43]. Also, one study examined the association between agility and artistry performance [17] (Table 4). There were no significant differences between qualifiers and non-qualifiers of the all-around competition in agility as measured by five consecutive 18 m shuttle sprints (running across the diagonal length of the gymnastics floor; 14 × 14 m) [24]. However, agility (five consecutive 18 m shuttle sprints) and sideways leg extension accounted for 43.7% of the variance of the score of “artistry” in the study of Kritikou et al. [17], and the authors assumed that repeated sprint ability was important for developing an “artistic image” through space and time. 

Twelve weeks of proprioceptive training improved coordination, agility (lateral agility and 20 yards test), and explosive strength in young rhythmic gymnasts [39], while small improvements were also observed in the control group in the lateral agility test. Non-specific tests, such as change in the direction speed tests, were used to evaluate agility in rhythmic gymnastics, and no study reported a sport-specific agility test with or without apparatus manipulation.

**Table 4:** Studies examining sprint speed and agility.

Sprint Speed									
		Participants							
Authors	Type of Study	EG (n)	CG (n)	Age (EG)	Age (CG)	Level	Test Used/Specific-Non-Specific	Study Outcome	Main Findings
Douda et al. (2007) [26]	CT	71 RG		8–10, 11–12 13–14 15–17		Low–High	Sprint speed 30 m/NS	Comparison between gymnasts’ levels and age categories. Training outcome.	RG athletes attained better scores in sprint speed compared to CG before intervention. Following 6 months of training, sprint speed in the RG athletes improved. Improvements were also observed in the CG in sprint speed, albeit to a lesser extent than in the EG
		81 non-gymnasts							
Douda et al. (2008) [6]	CS	15 elite		13.41 ± 1.62		Low–High	Sprint speed 30 m/NS	Comparison between gymnasts’ levels.	There was no difference between elite and non-elite gymnasts in sprint speed.
		19 non-elite							
Ivanova (2015) [47]	CS	16	27	10–12		-	“Temping” backbend test; bend running 30 m/sec test “zig-zag” skipping/NS	Comparison between gymnasts’ levels.	The EG outperformed the CG in the speed of the torso, the speed of lower limbs, and the speed of upper limbs. In addition, the speed of left arm was lower compared to the right arm. Speed abilities were characterized as low-level during that period of development.
Agility									
Agostini et al. (2017) [43]	RCT	15	15	15.4 ± 1.2	15.2 ± 1.5	Low–High	Square agility test/NS	Training outcome.	After 12 months, an improvement was observed in agility test performance in both groups, with a more significant improvement in the EG when compared with the CG. The addition of plyometric training to typical RG training improved agility in the EG.
Dobrijević et al. (2018) [39]	RCT	43	31	8 ± 0.8		Low	20-yard test; lateral agility test/NS	Training outcome.	Following 12 weeks of proprioceptive training, the EC demonstrated improvements in both agility tests, while improvements were also observed in the CG in the lateral agility test.
Donti et al. (2016) [24]	CS	24 Q		10.2 ± 1.0		Low–High	Five consecutive 18 m shuttle sprints/NS	Comparison between gymnasts’ levels.	There were no significant differences between qualifiers and non-qualifiers in agility test.
		22 NQ		9.7 ± 1.5					
Kritikou et al. (2017) [17]	CS	46		9.9 ± 1.3		High	Five consecutive 17 m shuttle sprints/NS	Association with performance.	Consecutive 17 m shuttle sprints significantly correlated with artistry score.

Note: NS, non-specific; S: specific; RCT, randomized controlled trial; CT, controlled trial; LS, longitudinal study; CS, cross-sectional study; BNT, Brazilian National Team; PNT, Portugal National Team; CG, control Group; EG, experimental group; Q, qualifiers; NQ, non-qualifiers.

### 3.7. Muscle Endurance

Muscular endurance is when less force is sustained over a longer period of time [24]. Eight studies examined muscular endurance in rhythmic gymnastics [6,17,20,21,24,26,41,48]. Two studies examined differences between gymnasts’ levels; three studies examined training interventions; three studies examined the association between muscle endurance and performance; one study examined age differences, and one study examined differences between gymnasts and controls (Table 5).

Muscular endurance was generally associated with rhythmic gymnastics performance scores, although some studies failed to detect differences between high and low-level gymnasts [6,24]. Training interventions improved muscular endurance more than rhythmic gymnastics training alone. Some muscle endurance capacities, but not all, seem to increase with age [41].

Muscular endurance of the back extensors and subscapular skinfold accounted for 29.2% (*p* < 0.01) of the variance in the “expression” score [17], and the authors assumed that the endurance of the back extensors was important for hyperextending the trunk in technical skills and regaining a firm standing position throughout a competitive routine [17]. In addition, moderate correlations were found between upper limb muscular endurance (i.e., push-ups) and technical execution score in non-elite youth gymnasts (i.e., 9–10 years) [24] and between muscular endurance and rhythmic gymnastics performances in 14–15- and 11–12 years old gymnasts [20,21]. Rhythmic gymnasts attained better scores in sit-ups compared to non-athletes (*p* < 0.001), while older gymnasts did not demonstrate better muscle endurance than younger athletes except for sit-up repetitions [41]. Longer-term interventions (> 6 months) with different training loads showed that gymnasts can increase their general (i.e., 30 repetitions of sit-ups) and specific muscular endurance, as well as sport-specific endurance of the lower limbs (“double rope jumping”) [20,21,26].

Notably, elite adolescent gymnasts could perform a similar number of sit-ups compared to non-elite [6], and the same was found for youth elite athletes (aged 9–12 years) [24], showing that this fitness attribute was not always improved according to gymnasts’ levels. General tests such as push-ups, sit-ups, back extensor repetitions, lifting legs, and a specific test (i.e., jumping into the rope with double turns) were used to assess muscle endurance and correlated with performances [17,24]. 

**Table 5:** Studies examining muscular endurance.

		Participants							
Authors	Type of Study	EG (n)	CG (n)	Age (EG)	Age (CG)	Level	Test Used Specific/Non-Specific	Study Outcome	Main Outcome
Cabrejas et al. (2022) [48]	RCT	23	22	10.52 ± 1.90	10.43 ± 1.78	High	Active straight leg raise test; bent knee fall out test; pelvic tilt test NS	Training outcome.	Following an 8-week functional core stability training, a trend in improving the performance of core stability was found with no significant differences between the EG and the CG.
Donti et al. (2016) [24]	CS	24 Q		10.2 ± 1.0		Low–High	One min push-ups; one min sit-up test; back extensors NS	Comparison between gymnast’s levels Association of fitness with performance	There were no significant differences between qualifiers and non-qualifiers in muscle endurance.
		22 NQ		9.7 ± 1.5					
Douda et al. (2007) [26]	CT	71 RG		8–10, 11–12 13–14 15–17		Low–High	Sit-ups	Training outcome.	RG athletes attained better scores in muscle endurance compared to CG before intervention. Following 6 months of training, muscle endurance improved in the RG athletes. No improvements were observed in the CG.
		81 non-gymnasts							
Douda et al. (2008) [6]	CS	15 elite		13.41 ± 1.62		High–Low	Sit-ups, NS	Comparison between gymnast’s levels. Association with performance.	There was no difference between elite and non-elite gymnasts in sit-ups repetitions.
		19 non-elite							
Gateva (2013) [41]	CS	120		11–19		Low–High	Sit-ups; back strength, NS	Comparison between gymnasts’ age categories.	Sit-ups increased with age. Back strength did not improve with age from 11–19 years old. Correlations were found between abdominal and back muscle endurance and between back muscles and lower-limb power.
Kritikou et al. (2017) [17]	CS	46		9.9 ± 1.3		High	Push-ups; sit-ups, NS	Association with performance.	Muscular endurance of the back extensors and subscapular skinfold accounted for 29.2% of the variance in the expression score.
Rutkauskaitė and Skarbalius (2012) [21]	CT	5 (A)		14.4 ± 0.55 A		-	Push-ups; sit-ups; lifting legs, NS	Training outcome.	Muscular endurance was significantly associated with performance scores in 14–15-year-old gymnasts. Following 48 weeks of intervention, significant improvements were found in muscular endurance. The training groups that involved more training sessions/week, more basic skills, and choreography elements outperformed the group with fewer sessions/week and fewer elements.
		5 (B)		14.2 ± 0.84 B					
Rutkauskaitė and Skarbalius (2009) [20]	CT	5 (A)		11–12		-	Push-ups; sit-ups; lifting legs; jumping into rope with double turns, S/NS	Training outcome.	Sports performance was associated with muscular endurance in 11–12-year-old gymnasts. Following 48 weeks of intervention, five different fitness programs were found efficient in improving muscular endurance and specific endurance, with no differences observed between training groups.
		5 (B)							
		5 (C)							
		5 (D)							
		5 (E)							

Note: S, specific; NS, non-specific; RCT, randomized controlled trial; CT, controlled trial; CS, cross-sectional study; CG, control group; EG, experimental group; A, group A; B, group B; C, group C; D, group D; E, group E; RG, rhythmic gymnastics; Q, qualifiers; NQ, non-qualifiers.

### 3.8. Balance

Balance is the ability to maintain the body’s center of mass above the base of support [49]. Dynamic balance reflects the motor control over the center of gravity in a continuously changing environment [50]. Nine studies examined balance in rhythmic gymnasts [17,20,24,46,49,50,51,52,53]. Two studies examined the association between balance and performance; three studies examined the effectiveness of training interventions; four studies examined differences between gymnasts’ levels; two studies examined age differences, and one study compared gymnasts from different sports (rhythmic, artistic, aerobic) (Table 6).

In the included studies, balance improved from childhood to adulthood and remained unaltered during puberty [49]. Also, balance was associated with technical execution as well as with other fitness attributes (i.e., flexibility, muscle strength) and with changes in body proportions due to growth and maturation [49]. In general, dynamic and static balances were higher in elite gymnasts compared to controls [50], while training interventions improved the ability to maintain balance [44] (Table 6).

Balance improves with age as sensorimotor systems mature [54]. For example, in the study of Poliszczuk et al. [49], it was reported that the ability to maintain dynamic balance increased from childhood to adolescence with no further improvement during puberty. Notably, associations were found between balance and changes in body proportions (i.e., height, weight, and somatotype) in adolescent gymnasts. One study reported that after four months of supplementary balance training, both the experimental and the control group improved static and dynamic balance; however, the gymnasts of the experimental group presented significantly higher changes in balance scores, thus underpinning the role of additional balance-specific training [53].

Although balance on the ball of the foot (a sport-specific test) was associated with artistry performance [17], some studies failed to detect differences between gymnasts of different competitive levels, probably because of the young age of the participants (9–10 years old) [24]. Adolescent rhythmic gymnasts demonstrated better static and dynamic balance compared to controls, and this difference was larger between older gymnasts with longer sport-specific experience [43,52]. In a cross-sectional study, Kyselovicova et al. [46] found a significant relationship between dynamic balance and isokinetic muscle strength, indicating the contribution of strength to balance performance. Specific (e.g., balance on the ball of the foot) and non-specific tests (e.g., Y-balance test) were used in the included studies, and both types correlated with gymnastics performance, indicating the importance of balance for this sport.

**Table 6:** Studies examining balance.

		Participants							
Authors	Type of Study	EG (n)	CG (n)	Age (EG)	Age (CG)	Level	Test Used/Specific-Non-Specific	Study Outcome	Main Findings
Calavalle et al. (2008) [51]	CS	15 RG		18.38 ± 4.57		High–Low	Stand in a bipedal postural configuration, barefoot, upright on the force platform/NS	Comparison between gymnasts’ levels.	RG gymnasts demonstrated higher balance values than students in the anteroposterior directions and lower in lateral distances. No differences were found between groups in the mean distance from the center of sway. In addition, gymnasts had the same strategy compared with controls in anteroposterior direction between 0.05 and 2 s. In long-time periods (>10 s), gymnasts demonstrated less stability than controls. Lastly, gymnasts showed a better strategy in lateral distances compared with controls, especially in mediolateral position.
		43 university students		22.09 ± 5.63					
Donti et al. (2016) [24]	CS	24 Q		10.2 ± 1.0		High–Low	Remain on the ball of the foot (“releve”) with their arms held above their head (third position) and the free foot at a low passe (fondue) for as long as possible/S	Comparison between gymnasts’ levels.	There were no significant differences between qualifiers and non-qualifiers in balance on the ball of the foot.
		22 NQ		9.7 ± 1.5					
Kioumourtzoglou et al. (1998) [52]	CS	20 group1		11–12		High	Dynamic balance; static balance/S-NS	Comparison between age categories. Association with performance.	In the group of 12–15-year-old gymnasts, dynamic balance, kinesthesis, and depth perception explained 56% of all-around skill. In 11–12-year-old gymnasts, eye–hand coordination, whole-body reaction time, and depth perception explained 40% of all-around skills.
		20 group2		13–14					
Kioumourtzoglou et al. (1997) [50]	CS	20 RG	20 ST	9–10		High	Static balance; dynamic balance/NS	Comparison between gymnasts’ levels and age categories.	Analysis showed that scores on measures of dynamic balance and static balance were higher for elite athlete groups (9–10, 11–12, and 13–15 years) than the corresponding control groups. Moreover, elite athletes in the oldest group (13–15 years) scored higher than those in the youngest group (9–10) in static balance.
		20 RG	20 ST	11–12					
		20 RG	20 ST	13–15					
Kritikou et al. (2017) [17]	CS	46		9.9 ± 1.3		High	“passé”/S	Association with performance.	Balance on the ball of the foot correlated with “artistry” and the “music and the movement” scores.
Kyselovičová et al. (2023) [46]	CS	6 RG		RG 17.7 ± 0.53		High	Y-balance test/NS	Comparison between different sports.	Significant differences between groups in the composite score of the Y-balance test of the dominant and non-dominant symmetry were found. In addition, there was a significant association between isokinetic dominant limp extension strength and Y-balance test in RG.
		5 AR		AR 14.4 ± 5.92					
		7 AE		AE 15.87 ± 0.73					
Palomares et al. (2019) [53]	RCT	30	30	15.4 ± 1.2	15.2 ± 1.5	High-Low	Balance test in the Arabesque, backgrab, and heel stretch positions. Movement from the arabesque to the passé position, S.	Training outcome. Comparison between gymnasts’ levels.	Following 16 weeks of a “conjugate influence method” of training, both groups (high and low-level gymnasts) presented improvements in static and dynamic balance; however, the gymnasts in the experimental group presented significantly higher mean scores in all the tests than those in the control group.
Poliszczuk et al. (2012) [49]	LS	13		9.79 ± 1.41		-	Posturography/NS	Training outcome.	After two years of RG training, significant improvements were found in dynamic balance indicators in RG aged 7–12.
				11.19 ± 1.4					
				12.1 ± 1.51					
Rutkauskaitė and Skarbalius (2009) [20]	CT	5 (A)		11–12		-	Test of “leg keeping”/S	Training outcome	Following 48 weeks of intervention, five different fitness programs were similarly efficient in improving balance with no differences between training groups.
		5 (B)							
		5 (C)							
		5 (D)							
		5 (E)							

Note: S, specific; NS, non-specific; RCT, randomized controlled trial; CT, controlled trial; LS, longitudinal study; CS, cross-sectional study; CG, control group; EG, experimental group; A, group A; B, group B; C, group C; D, group D; E, group E; ST, students; RG, rhythmic gymnastics; AR, artistic gymnastics; AE, aerobic gymnastics; Q, qualifiers; NQ, non-qualifiers.

### 3.9. Coordination

Coordination is the ability to execute a sequence of movements smoothly and accurately repeatedly [49]. Eight studies examined coordination in rhythmic gymnasts [20,21,39,50,52,55,56,57]. Four studies examined training outcomes; two studies compared different competitive levels; three studies compared different age categories, and three studies examined the association between coordination and performance (Table 7). In general, high-level gymnasts demonstrated better whole-body coordination than low-level gymnasts or controls [50]. Following intervention, gymnasts improved their coordination compared to controls [55], irrespective of age [20,21]. In some cases, both the intervention and the control groups that followed typical rhythmic gymnastics training improved their coordination abilities, thus suggesting that coordination also improved following sport-specific training [55].

Coordination was considered an important performance determinant in a group of 127 junior and senior rhythmic gymnasts (R2 = 0.38) when gymnasts were examined as one group [56]. Coordination was also the strongest determinant of performance in the advanced (R2 = 0.42) and intermediate-level gymnasts (R2 = 0.50), compared to the elementary-level gymnasts, probably because of the higher demands in complex skill execution [56]. In elite junior gymnasts, the proportion of the variance explained in gymnastics performance scores by “two-hand coordination” and “aiming” scores was 73.6% in hoop and 65.7% in the clubs [57]. “Two-hand coordination” and “selective attention” explained 43.7% of the variance in ball performance, while performance in ribbon was predicted only by “two-hand coordination” (13.4%) [57]. 

In the studies examined, both general tests (i.e., eye-hand coordination, whole-body coordination, two-hand coordination, and line tracking) and specific tests were used (i.e., ball rolling over the arms, jumping through a rope, skipping through a hoop, and club juggling), again indicating the importance of coordination for rhythmic gymnastics performance.

**Table 7:** Studies examining coordination.

		Participants							
Authors	Type of Study	EG (n)	CG (n)	Age (EG)	Age (CG)	Level	Test Used/Specific-Non-Specific	Study Outcome	Main Findings
Ahmed (2016) [55]	CT	10	10	9.69 ± 0.382		Low	Ability to accurately determine the status, control the movement rhythm, control balance, motor control, ability of reaction speed/NS	Training outcome	Following an 8-week intervention of coordination training, coordination abilities were significantly higher in the EG compared with the CG. In addition, performance scores in the clubs, hoop, rope, and ball were significantly higher in the EG compared with the CG. Nevertheless, two coordination capacities (reaction speed and motor organization) and performance scores in rope and clubs were also improved in the CG from baseline.
Dobrijević et al. (2018) [39]	RCT	43	31	8 ± 0.8		Low	Twisting/agility in the air; “figure eight” with bending and jumping; jumping over and pulling under/NS	Training outcome.	Following 12 weeks of proprioceptive training no differences were observed between the EG and the CG, showing that coordination also improved following RG training.
Giannitsopoulou et al. (2003) [57]	CS	33 young juniors		11–12		High (National Team)	Two-hand coordination; line tracking; wrist–finger dexterity/NS	Comparison between different age categories. Association with performance.	Different coordination abilities correlated with performance in the two age groups (juniors, young juniors). In junior gymnasts, the amount of performance variance explained by two-hand coordination and aiming was 73.6% in hoop and 65.7% in clubs. Two-hand coordination and selective attention explained 43.7% of variance in ball performance, while performance in ribbon was predicted only by two-hand coordination (13.4%). In young junior gymnasts (11–12 years), the only significant predictor of performance was memory (grouping) and choice reaction time, which explained 18.5% of variance in ball performance.
		11 juniors		13–14					
Kioumourtzoglou et al. (1998) [52]	CS	20 group 1		11–12		High	Depth perception; eye–hand coordination; kinesthesis; whole-body coordination; Lafayette instruments (dynamic balance); rope, hoop, ball, and all-around scores/NS-S	Comparison between different age categories. Association with performance.	In the youngest group of gymnasts (11–12 years old), eye–hand coordination, whole-body reaction time, and depth perception explained 40% of the all-around skill. In the oldest group of gymnasts (13–15 years), depth perception kinesthesis and dynamic balance correlated with performance.
		20 group 2		13–14					
Kioumourtzoglou et al. (1997) [50]	CS	20 RG	20 ST	9–10		High	Whole-body coordination; kinesthesis; eye–hand coordination; perceptual abilities/NS-S	Comparison between gymnasts’ levels and different age categories.	Analysis showed that scores of whole-body coordination were higher for the elite groups of athletes (aged 9–15 years) than for corresponding control groups. Moreover, elite athletes in the oldest group (13–15 years) scored higher than those in the youngest group (9–10 years) in anticipation of coincidence and eye–hand coordination. These findings indicate the presence of systematic differences between elite athletes and non-athletes in motor abilities related to this sport.
		20 RG	20 ST	11–12					
		20 RG	20 ST	13–15					
Purenović-Ivanović et al. (2016) [56]	CS	22 beginners		8.04 ± 0.75		Low–High	Ball rolling over the arms; throwing, catching, jumping through a rope, skipping through a hoop club, juggling/S	Comparison between gymnasts’ levels. Association with performance.	Specific coordination skills are associated with performance only in the group of advanced and intermediate gymnasts but not in the beginner group. Hoop skipping and club juggling were the best predictors of performance scores in the total sample.
		39 intermediates		10.09 ± 0.81					
		26 advanced		12.25 ± 0.89					
		25 juniors		14.53 ± 0.74					
		15 seniors		17.53 ± 1.37					
Rutkauskaitė and Skarbalius (2012) [21]	CT	5 (A)		14.4 ± 0.55 A		-	Ten s running into the rope/S	Training outcome.	Following 48 weeks of intervention, significant improvements were found in coordination abilities, with no differences observed between different training groups.
		5 (B)		14.2 ± 0.84 B					
Rutkauskaitė and Skarbalius (2009) [20]	CT	5 (A)		11–12.		-	Electronic indicator of the error of movement/NS	Training outcome.	Following 48 weeks of intervention, five different fitness programs were efficient in improving coordination, with no differences observed between training groups.
		5 (B)							
		5 (C)							
		5 (D)							
		5 (E)							

Note: S, specific; NS, non-specific; RCT, randomized controlled trial; CT, controlled trial; CS, cross-sectional study; CG, control group; EG, experimental group; A, group A; B, group B; C, group C; D, group D; E, group E; RG, rhythmic gymnastics; Q, qualifiers; NQ, non-qualifiers; ST, students.

## 4. Discussion

The main finding of this study was that performance scores in rhythmic gymnastics were associated with specific fitness parameters (i.e., flexibility, aerobic capacity, lower-limb muscle power, agility, muscular endurance, balance, and sport-specific coordination), and this association was observed from a young age. It was also found that high-level gymnasts outperformed low-level gymnasts and controls in some but not all the fitness parameters examined. For example, high-level gymnasts scored higher in flexibility, aerobic capacity, and muscle power. However, no differences were observed between gymnasts’ levels in sprint speed, agility, muscular endurance, and balance. Older gymnasts also demonstrated higher scores compared to younger gymnasts in flexibility, aerobic capacity, balance, and sport-specific coordination but not in muscle endurance, while some studies even showed a decline in muscle power with age. Training intervention studies are sparse, and although rhythmic gymnastics training may also improve specific fitness parameters to some extent, systematic training always induced larger gains in flexibility, lower-limb muscle power, muscular strength, agility speed, muscular endurance, balance, and coordination. Notably, muscular strength, speed, and agility were largely under-researched in rhythmic gymnastics. 

This scoping review confirmed the importance and the association of flexibility as a major performance determinant in rhythmic gymnastics. However, it has been shown that excessive flexibility training and a deficit in muscular strength resulted in training errors [36]. Uncontrolled and excessive lumbar extension/flexion, forcing “turnout” from a very young age, and high mechanical loads have been identified as the most common training mistakes leading to musculoskeletal overload and injury [58]. In addition, the lack of balance between active and passive flexibility actions may predispose youth gymnasts to specific (i.e., spine) injuries. Certain aspects of growth and maturation also predispose youth athletes to injuries involving the immature spine (e.g., risk for spondylolysis, spondylolisthesis). Importantly, low-back pain complaints are very common in female rhythmic gymnasts (86%) [59,60] due to the large tension placed on spine structures from the combination of excessive back extension and lack of strength [60]. Furthermore, because of the unilateral character of technical skills and the large number of skill repetitions, apparent asymmetries are observed that increase the risk for injury or pathology (i.e., scoliosis) [61]. There are also evident deficiencies in coach, athlete, and parent knowledge and behaviors regarding injury prevention programs in youth rhythmic gymnastics populations despite evidence supporting their implementation [62], and it is reported that gymnasts continue training despite experiencing pain [63].

Aerobic fitness was also a strong correlate of performance scores in rhythmic gymnastics, discriminating elite from non-elite athletes. Competitive routines last about 60 to 90 s and combine high-intensity technical elements with a dexterous manipulation of the apparatus. Continuous high-intensity exercise for 60 to 90 s requires considerable contribution to aerobic metabolism [32]. Increased aerobic fitness also allows gymnasts to tolerate the extreme demands of training loads (two–three training sessions per day) and competitions and may enhance recovery between sessions, thus reducing injury risk in developing athletes. Nevertheless, the studies on aerobic capacity are limited, and only one study examined differences between developing gymnasts, while no training studies were found. Thus, despite the importance of cardiovascular fitness for gymnasts’ health and performance, there is a lack of evidence for optimizing aerobic training in rhythmic gymnastics, and further studies are needed.

Gymnasts perform jumps, leaps, and hops of progressive difficulty while moving through space (14 × 14 m) for an extended period (60–90 s). The results of this review revealed an interesting finding: lower-limb muscle power discriminated high from low-level gymnasts only when performance was assessed by measuring technical leaps and jumps. In contrast, the studies using the all-around score to assess performance showed no association with muscle power. This shows that lower-limb muscle power is significantly associated with the performance of technical leaps and jumps (i.e., with specific technical skills) and not with the overall rhythmic gymnastics performance scores. From the muscle power tests used (e.g., countermovement jump, squat jump, hopping test), the hopping test ground contact time (which is related to muscle stiffness) was significantly correlated with technical leap execution. In addition, the height of the hopping test was significantly higher in elite than sub-elite gymnasts, probably showing more effective use of the stretch-shortening cycle as gymnasts become more powerful. When jump height was normalized to Body Mass Index, muscle power declined with age due to an increase in body size without simultaneous gains in strength [42]. This finding has important implications for the design of developmentally appropriate training interventions by coaches and practitioners. The limited number of training interventions (n = 7) showed that rhythmic gymnastics training alone was also effective in improving lower-limb muscle power, albeit always to a lesser degree compared with additional resistance training, indicating the need to include supplementary strength training in developing gymnasts. 

The results of this scoping review revealed that the physical activity components of strength, speed, and agility are largely under-researched in rhythmic gymnastics (two, three, and four studies, respectively). Of these studies, only one study included a strength training intervention; one included speed training, and two studies included agility training. Thus, guidelines for age-appropriate muscular fitness development programs in rhythmic gymnastics do not exist in the literature despite the high training loads to which gymnasts are subjected. The fact that no differences were observed between gymnasts’ levels in sprint speed, agility, muscular endurance, and balance and that muscle strength, sprint speed, and agility are largely under-researched in rhythmic gymnastics may probably underpin inadequate training practices. In addition, older gymnasts did not outperform younger gymnasts in muscle endurance, and some studies even showed a decline in muscle power with age as gymnasts grew and their relative strength decreased.

To counteract inadequate training practices, long-term athlete development models suggest that muscular strength and power should be developed throughout childhood and adolescence with an optimal time frame between 12–17 years [11,64]. This constitutes a challenge for coaches and practitioners in early specialization sports, who should include multiple modes of training aiming to develop strength and power, along with several other components of fitness throughout childhood and adolescence [65]. There is evidence to suggest that bodyweight plyometric training, traditional strength training using external resistance, or a combination of the two should be implemented as early as possible [66]. Although more mature children may demonstrate greater gains in strength, younger children following developmentally appropriate resistance training achieve meaningful improvements in strength, which are transferred to locomotion skills such as running and jumping [67]. An increase in strength improves power generation [44], technical performance [41], and the speed at which competitive skills are executed [48,68]. 

It should be noted that it is a common practice in rhythmic gymnastics to use conditioning programs that are skill-driven due to the specific demands of the sport [69]. Although training specificity cannot be underestimated, research demonstrates that participating in sport alone without the addition of supplementary strength training fails to optimize athletic development [67] and may increase the risks of overuse injuries due to the repetitive loading on weak and immature musculoskeletal structures in the absence of sufficient recovery [7]. Overuse injuries concerning joint surfaces (i.e., osteochondritis dissecans) and traction apophysitis (i.e., Osgood–Schlatter disease, Sever’s disease) are notably pervasive with youth athletes, who proceed too rapidly to higher levels of training and competition [62]. A safe and effective strength training program for preadolescent athletes requires the consideration of many variables, such as the age at which a child should begin strength or resistance training, the number of sets and repetitions that should be performed, the recovery intervals between sets, and the weekly training frequency. Due to the current lack of longitudinal and well-controlled studies on fitness development in rhythmic gymnastics and especially on muscular strength, power, speed, and agility, further research is required. 

Although this study is the first attempt to map the broad field of physical fitness in rhythmic gymnastics, there are some limitations that should be mentioned. Due to the very large heterogeneity between studies (RCTs, CTs, CS, LS), effect sizes for the intervention studies were not presented, and it was impossible to calculate the risk of bias. In addition, the competitive level was defined as “high” and “low”, referring, in general, to all the gymnasts of higher or lower competitive level and training experience, irrespective of whether the “high-level” gymnasts were National team members or youth qualifiers of the all-around. Similarly, the terms ”younger” and “older” gymnasts are used to describe a general age trend in fitness development and not specific differences between child, adolescent, and adult gymnasts. 

## 5. Conclusions 

In conclusion, a better understanding of the training process and progression, along with the interactions of training with growth and maturation, may optimize the design of training programs, making them more effective and also safer and more enjoyable by reducing injury risk and enhancing gymnasts’ health and well-being. Research on training interventions in developing gymnasts is limited, and guidelines for training protocols are unclear. This is a critical issue in rhythmic gymnastics as many girls worldwide take part in this sport and are subjected to lengthy and sometimes excessive training and competition demands from a very young age.

## Data Availability

All the data are made public in the Tables.

## Supplementary Materials

The following supporting information can be downloaded at https://www.mdpi.com/article/10.3390/sports12090248/s1, File S1 36Preferred Reporting Items for Systematic reviews and Meta-Analyses extension for Scoping Reviews (PRISMA-ScR) Checklist; File S2: PRISMA Flow Diagram for the scoping review process.
