# Supplementary material for: The Importance of Physical Fitness Parameters in Rhythmic Gymnastics: A Scoping Review
Source: Sports (Basel). 2024 Sep 7;12(9):248. doi: 10.3390/sports12090248 (PMC11435787; doi:10.3390/sports12090248)
Supplement: Supplementary file 1 [file sports-12-00248-s001.zip › R1_Supplementary File_2_S2.pdf]

PRISMA Flow Diagram for the scoping review process

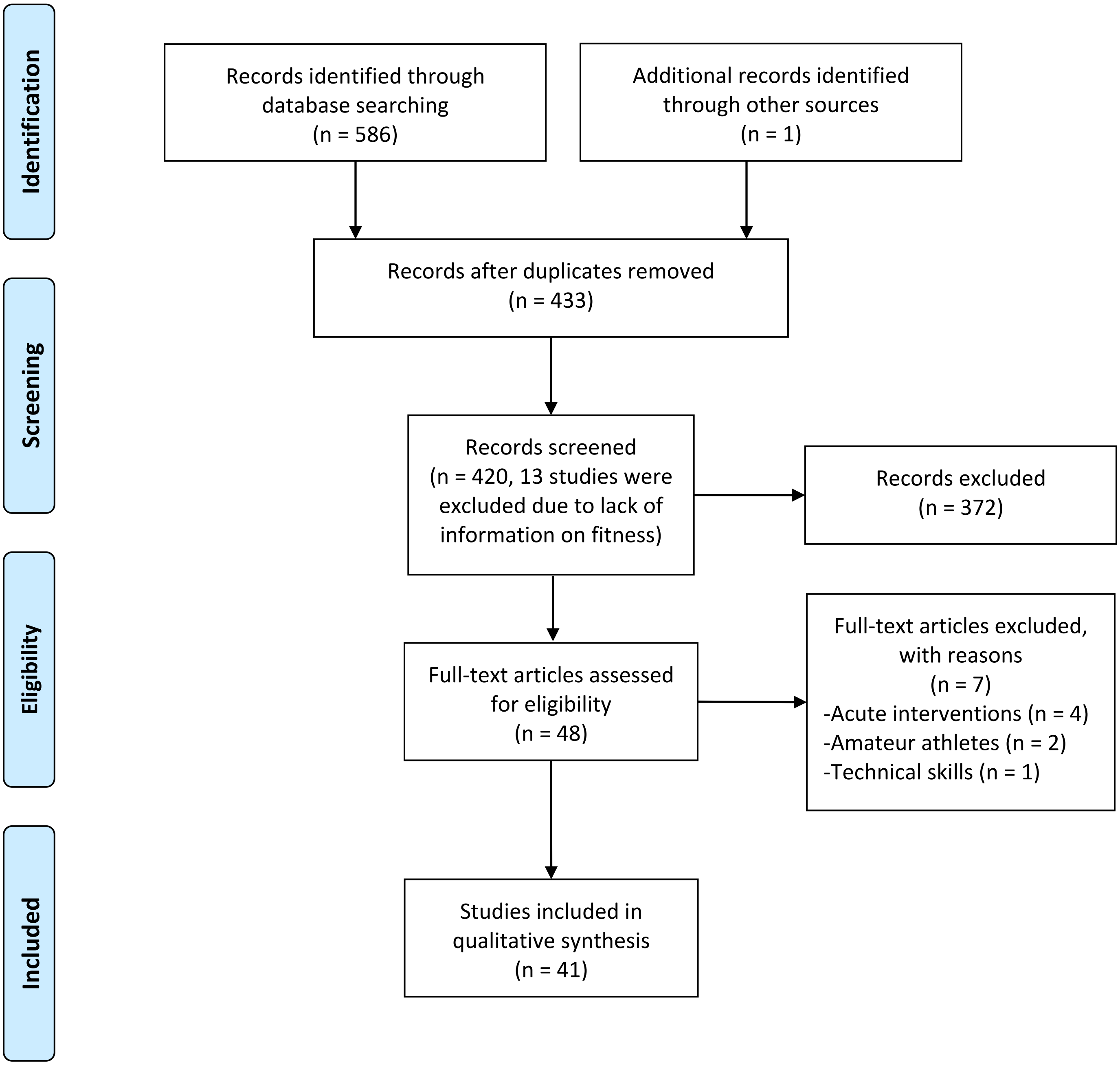

From: Moher D, Liberati A, Tetzlaff J, Altman DG, The PRISMA Group (2009). *P*referred *R*eporting *I*tems for *S*ystematic Reviews and *M*eta-*A*nalyses: The PRISMA Statement. PLoS Med 6(6): e1000097. doi:10.1371/journal.pmed1000097

For more information, visit www.prisma-statement.org.
